# Current News

**Published:** 1898-05

**Authors:** 


### Current News.

### MASSACHUSETTS DENTAL SOCIETY.

The next Annual Meeting of the Massachusetts Dental Society will be held at Mechanics Building, Huntingdon Avenue, Boston, Wednesday and Thursday, on the 1st and 2d of June next, and promises to be one of the most successful in the history of the association.

The committee having the matter in charge hope to secure one of the largest exhibits of dental depot supplies, dental laboratory work, chemicals, and other goods necessary or applicable to the practice of dentistry which has never been shown in New England.

To this end they have secured a large, well-lighted room for the accommodation of exhibitors, and have formulated such rules and regulations to govern the distribution of space and the admission of exhibits as will insure just and fair treatment of all, whether the amount of space they require shall be great or otherwise.

*The cost of space* will be seventy-five cents per running foot (not less than eight feet will be sold to any one exhibitor), and the width of the exhibit shall not be greater than six feet.

*Payment for space* must be made in full by the 20th of May next. (Checks, etc., to be made payable to the Chairman of the Committee on Halls and Exhibits.) A small extra charge will be made for electricity to those exhibiting engines, lathes, furnaces, etc.

*Choice of space* will be sold at auction to the highest bidder. The committee fail to see how there can be much choice of space in the rooms they have taken, but have introduced this provision as a safeguard both to themselves and to the exhibitors.

Exhibitors will not be permitted to have any nostrums on their tables, neither will they be allowed to make this a bargain sale of bankrupt stock, but are expected to come to us with their regular lines of goods, of which they can sell as much as they please as cheaply as they like.

The committee reserve the right to reject or eject any applicant for space, or occupant of the same, whenever it shall appear to them to be in the interest of the Society so to do.

The committee will have full charge and control of the exhibits.

Hoping to have the pleasure of marking off a large block of space for your house,

We remain, respectfully,

F. S. Belyea, *Chairman*,
W. E. Boardman,
J. W. Bailey,
*Committee on Halls and Exhibits.*

Brookline, Mass., April 1, 1898.

---

### NATIONAL DENTAL ASSOCIATION, DIVISION OF THE EAST.

At the request of William Jarvie, V. H. Jackson, W. W. Walker, of New York; S. G. Watkins, B. F. Luckey, of New Jersey; E. T. Darby, D. N. McQuillan, of Pennsylvania; L. D. Shepard, of Massachusetts; H. A. Smith, of Ohio; and G. E. Hunt, of

Indiana, a meeting of the members of the National Dental Association residing in the East is called at Odd Fellows' Temple, Albany, N. Y., on Thursday, May 12, 1898, at two o'clock, to organize a branch of the National Dental Association, and to transact any other business which may properly come before them.

As this meeting is coincident with that of the New York State Dental Society, any member obtaining a certificate when he purchases his railroad ticket will be entitled to reduced return fare.

Reduced rates at Kenmore Hotel.

THOMAS FILLEBROWN,
*President National Dental Association.*
JAMES MACMANUS,
*Vice-President National Dental Association for the East.*

---

### HARVARD ODONTOLOGICAL SOCIETY.

THE following are the officers elected for the Harvard Odontological Society for 1898:

President, Arthur H. Stoddard, D.M.D., 196 Marlboro Street, Boston; Recording Secretary, Joseph T. Paul, D.M.D., 3 Park Street, Boston; Corresponding Secretary, Edward B. Hitchcock, M.D., D.M.D., Newton, Mass.; Treasurer, Lyman F. Bigelow, D.M.D., 196 Marlboro Street, Boston; Editor, Henry L. Upham, D.M.D., 128 Charles Street, Boston.

*Executive Committee.*—Joseph T. Paul, D.M.D., Frank T. Taylor, D.M.D., William P. Cooke, D.M.D.

The Twentieth Annual Meeting is to occur February 26, with a banquet, admitting ladies, at Young's Hotel.

E. B. HITCHCOCK,
*Corresponding Secretary.*

